# Ang2 inhibitors and Tie2 activators: potential therapeutics in perioperative treatment of early stage cancer

**DOI:** 10.15252/emmm.201708253

**Published:** 2021-06-14

**Authors:** Kabir A Khan, Florence TH Wu, William Cruz‐Munoz, Robert S Kerbel

**Affiliations:** ^1^ Department of Medical Biophysics University of Toronto Toronto ON Canada; ^2^ Biological Sciences Platform Sunnybrook Research Institute Toronto ON Canada

**Keywords:** adjuvant, angiogenesis, immunotherapy, metastasis, neoadjuvant, Cancer, Vascular Biology & Angiogenesis

## Abstract

Anti‐angiogenic drugs targeting the VEGF pathway are most effective in advanced metastatic disease settings of certain types of cancers, whereas they have been unsuccessful as adjuvant therapies of micrometastatic disease in numerous phase III trials involving early‐stage (resectable) cancers. Newer investigational anti‐angiogenic drugs have been designed to inhibit the Angiopoietin (Ang)‐Tie pathway. Acting through Tie2 receptors, the Ang1 ligand is a gatekeeper of endothelial quiescence. Ang2 is a dynamically expressed pro‐angiogenic destabilizer of endothelium, and its upregulation is associated with poor prognosis in cancer. Besides using Ang2 blockers as inhibitors of tumor angiogenesis, little attention has been paid to their use as stabilizers of blood vessels to suppress tumor cell extravasation and metastasis. In clinical trials, Ang2 blockers have shown limited efficacy in advanced metastatic disease settings. This review summarizes preclinical evidence suggesting the potential utility of Ang2 inhibitors or Tie2 activators as neoadjuvant or adjuvant therapies in the prevention or treatment of early‐stage micrometastatic disease. We further discuss the rationale and potential of combining these strategies with immunotherapy, including immune checkpoint targeting antibodies.

## Graphical abstract

In light of the latest preclinical evidence, R. Kerbel and colleagues discuss in this review our current understanding of the effects of targeting the Ang/Tie pathway in the context of cancer angiogenesis and metastasis.

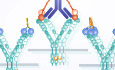

**Glossary**
Adjuvant therapy
  Treatment given to patients soon after surgical resection of tumors, when there is no evidence of distant macroscopic metastases, but who have micrometastases.
Cancer immunotherapy
  Therapeutics given to re‐invigorate or stimulate an immune response against cancer. This can be in the form of adoptive transfer of T cells, vaccines, or more commonly, the use of immune checkpoint inhibiting antibodies.
Metastatic cascade
  The stepwise events in which tumor cells leave the site of a primary tumor and establish tumors at new sites (metastases).
Micrometastatic disease
  Small metastases that can be detected by microscopy, but not by conventional radiologic imaging methods.
Neoadjuvant therapy
  Treatment given to patients before surgical resection of tumors.
Perioperative therapy
  Therapeutics administered around the time of surgery, including both neoadjuvant and/or adjuvant therapy.

## Introduction

In oncology, anti‐angiogenic drugs, especially those targeting the vascular endothelial growth factor (VEGF) pathway, were originally designed to inhibit the development of tumor blood vessels (“sprouting angiogenesis”) that supply growing tumors with oxygen and nutrients (Folkman, 2007; Jayson *et al,* 2016), as well as various tumor growth‐stimulating factors (“angiocrines”) secreted by endothelial cells (Pasquier *et al,* 2020). Interest in anti‐angiogenic drugs, in particular, those that target the VEGF pathway, has recently been reinvigorated with several recent approvals when such drugs are used in combination with antibodies that target the immune checkpoint PD‐1/PD‐L1 pathway. Indeed, there is growing preclinical and clinical evidence that such anti‐angiogenic agents have immunomodulatory effects by virtue of blocking the immunosuppressive effects of angiogenic factors such as VEGF as well as angiopoietin‐2 (Ang2) (Motz & Coukos, 2011; Fukumura *et al,* 2018; Khan & Kerbel, 2018; Rahma & Hodi, 2019). In this review, we discuss three main therapeutic concepts and their possible use for perioperative treatment or prevention of early‐stage metastatic disease; these include (i) specific inhibition of Ang2 without inhibition of Ang1, (ii) Ang1 supplementation, and (iii) selective Tie2 activation.

## VEGF/VEGFR2 pathway inhibitors in oncology

The VEGF signaling pathway has been identified as the major driver of tumor angiogenesis (Ferrara, 2002; Ferrara & Kerbel, 2005; Kerbel, 2008; Apte *et al,* 2019), and upregulation of the soluble VEGF‐A ligand (often simply referred to as VEGF) is associated with a wide range of malignancies (Kut *et al,* 2007). All currently approved anti‐angiogenic drugs thus target either VEGF or its main endothelial cell surface signaling receptor, VEGF receptor 2 (VEGFR2) (Ebos *et al,* 2009b; Jayson *et al,* 2016; Apte *et al,* 2019). From 2004 to 2021, at least fourteen VEGF/VEGFR2‐targeted agents (bevacizumab, ramucirumab, aflibercept, sunitinib, sorafenib, pazopanib, axitinib, regorafenib, vandetanib, nintedanib, lenvatinib, cabozantinib, fruquintinib, and tivozanib) have successfully gained regulatory approval around the world as cancer treatments in a variety of indications, including colorectal, lung, breast, renal, brain, ovarian, cervical, hepatic, and thyroid carcinomas, as well as pancreatic neuroendocrine tumors and soft‐tissue sarcomas (Shirley, 2018; Zirlik & Duyster, 2018). These approvals are all based on randomized phase III clinical trial results in late‐stage advanced metastatic disease treatment settings.

In contrast, these same agents have not been successful in the corresponding early‐stage disease settings of the same cancers (Table 1). For instance, bevacizumab, which is approved for treating metastatic colorectal cancer (CRC) in combination with chemotherapy, has failed in the adjuvant therapy setting (i.e., given after surgery with curative intent) to improve disease‐free survival in four phase III trials involving resectable CRC. In these clinical trials, bevacizumab was given with chemotherapy followed by a period of maintenance therapy where it was administered alone (de Gramont *et al,* 2012; Allegra *et al,* 2013; Midgley *et al,* 2014; Benson *et al,* 2016). Sunitinib, which is approved for treating metastatic renal cell carcinoma (RCC), has failed as adjuvant therapy to improve overall survival (OS) in two phase III trials involving resectable RCC (Haas *et al,* 2016; Ravaud *et al,* 2016). See Table 1 for more examples, e.g., breast cancer. In some trials, there initially appeared to be transient benefits that eventually faded over time after discontinuation of adjuvant therapies (de Gramont *et al,* 2012; Allegra *et al,* 2013; Haas *et al,* 2016). As a result, there is now a heightened awareness that the biology of macrometastases and micrometastases can be very different with respect to therapeutic responsiveness (Duensing & Hohenfellner, 2016; Saltz, 2016), as some forewarned (Grothey, 2007; Schneider & Sledge, 2009). In response, and echoing similar comments by us and other preclinical investigators (Ebos *et al,* 2009a; Ebos & Kerbel, 2011; Vasudev & Reynolds, 2014), some clinical investigators recommend more detailed and relevant preclinical modeling of therapy for micrometastatic disease (and postsurgical treatment settings) to more accurately predict drug candidates that will have the best chance to succeed in the clinic (Cameron *et al,* 2013).

**Table 1:** Randomized phase III clinical trials of VEGF pathway inhibitors as adjuvant (Postoperative) cancer therapies.

Trials	Cancer Type	Arms	Results associated with VEGF Pathway Inhibition
NSABP C‐08^a^ AVANT*^b^ QUASAR2^c^ E5204^d^	Colorectal cancer	Chemotherapy +/− bevacizumab	No DFS or OS benefit (*AVANT: worse OS with Bev)
BEATRICE^e^ ECOG‐5103^f^ BETH^g^	Breast cancer	Chemotherapy +/− bevacizumab	No IDFS or OS benefit
AVAST‐M^h^	Melanoma	Observation vs. bevacizumab	DFI benefit, but no OS benefit
E1505^i^	Non‐small‐cell lung carcinoma	Chemotherapies +/− bevacizumab	No DFS or OS benefit
STORM^j^	Hepatocellular carcinoma	Placebo vs. sorafenib	No RFS/DFS or OS benefit
ASSURE^k^ S‐TRAC^l^	Renal cell carcinoma	Placebo vs. sunitinib	No DFS or OS benefit
ATLAS^m^	Renal cell carcinoma	Placebo vs. axitinib	No DFS benefit, except in highest risk sub‐population
PROTECT^n^ ECOG‐ACRIN‐2810^o^	Renal cell carcinoma	Placebo vs. pazopanib	No DFS benefit
SORCE^p^	Renal cell carcinoma	Placebo vs. sorafenib	No DFS or OS benefit

DFI, disease‐free interval; DFS, disease‐free survival; IDFS, invasive DFS; OS, overall survival; RFS, recurrence‐free survival.

^a^ Allegra *et al* (2013).

^b^ de Gramont *et al* (2012), André *et al* (2020).

^c^ Kerr *et al* (2016).

^d^ Benson *et al* (2016).

^e^ Cameron *et al* (2013).

^f^ Miller *et al* (2014, 2018).

^g^ Slamon *et al,* (2013).

^h^ Corrie *et al* (2014, 2017).

^i^ Wakelee *et al* (2016, 2017).

^j^ Bruix *et al* (2014).

^k^ Haas *et al* (2016).

^l^ Ravaud *et al* (2016).

^m^ Gross‐Goupil *et al* (2018).

^n^ Motzer *et al* (2017).

^o^ Appleman *et al* (2019).

^p^ Eisen *et al* (2020).

## Inhibiting the angiopoietin‐Tie2 pathway in advanced metastatic disease

In recent years, a different class of drug developed as anti‐angiogenic agents have been assessed which target the Angiopoietin‐Tie pathway (Table 2). This pathway consists of two tyrosine kinase cell surface receptors Tie1 and Tie2 and three soluble ligands Angiopoietin‐1, 2, and 4 (Ang1, Ang2, and Ang4 in humans) (Saharinen *et al,* 2017). The Tie2 receptor is widely expressed on the surface of resting endothelial cells and is also expressed by a subset of hematopoietic cells (e.g., tumor‐infiltrating myeloid cells) (Dumont *et al,* 1992; De Palma *et al,* 2005; Huang *et al,* 2010), as well as pericytes, albeit at lower levels than endothelium (Teichert *et al,* 2017). Acting on Tie2^+^ endothelial cells, the Ang1 ligand maintains vessel stability and endothelial cell survival during development (Kim *et al,* 2000; Koh, 2013). While Ang1 is not essential for maintaining stability of quiescent adult vessels in baseline conditions (in the absence of cancer or pathology), it does play a role in modulation of the adult vasculature post‐injury where it limits aberrant angiogenic tissue responses (Jeansson *et al,* 2011). Angiopoietin‐2 (Ang2) expression is increased and released by endothelial cells during vascular remodeling and inflammation (Huang *et al,* 2010). Ang2 serves as an autocrine early initiator of angiogenesis, where it first de‐stabilizes quiescent blood vessels, thus allowing VEGF to drive proliferation and chemotactic migration of angiogenic vessel sprouts (Huang *et al,* 2010). Moreover, Ang2 also activates Tie2‐expressing monocytes/ macrophages (TEMs), which have their own pro‐angiogenic, pro‐tumorigenic, pro‐metastatic, and immunosuppressive functions (Huang *et al,* 2010; De Palma & Naldini, 2011; Ibberson *et al,* 2013). There have also been reports of Ang2 expression by tumor cells (Koga *et al,* 2001; Sfiligoi *et al,* 2003), and more recently, this has been shown to be the case for metastatic melanoma, where tumor Ang2 expression has effects on enhancing metastasis and protecting cells from oxidative stress, independent of its effects on immune cells or the vasculature (Abdul Pari *et al,* 2020).

**Table 2:** Angiopoietin/Tie2 pathway modulators in development for oncological use.

Agent	Mechanism	Furthest Stage in Development
AMG386/ trebananib (Amgen)	Peptide‐Fc fusion ‘peptibody’ against Ang2 & Ang1	Phase III trials in Advanced Ovarian Cancer: TRINOVA‐1—↑PFS; no OS^ITT^ benefit^a^; TRINOVA‐2—no PFS benefit^b^; TRINOVA‐3—PFS benefit unlikely^c^, terminated [NCT01493505] TRINOVA‐3—OS benefit in subgroup of patients with ascites^d^.
AMG780 (Amgen)	Fully human IgG2 mAb against Ang2 & Ang1	Phase I trial in Advanced Solid Tumors: Part A for dose escalation—completed^e^; Part B for dose expansion—terminated [NCT01137552]
CVX‐060 (Pfizer)	‘CovX‐body’ against Ang2	Phase II trials in Metastatic Renal Cell Carcinoma: with axitinib—terminated [NCT01441414]; with sunitinib—terminated [NCT00982657]
MEDI‐3617 (MedImmune)	Fully human IgG1κ mAb against Ang2	Phase I trial in Metastatic Melanoma with anti‐CTLA‐4 (tremelimumab)—active, not recruiting [NCT02141542]
REGN910/ nesvacumab (Regeneron)	Fully human IgG1 mAb against Ang2	Phase I trials in Advanced Solid Tumors alone—completed^f^; with ziv‐aflibercept (anti‐VEGF‐A/VEGF‐B/PlGF)—results pending^g^
LY3127804 (Eli Lilly)	Humanized IgG4 mAb against Ang2	Phase I trial in Advanced Solid Tumors alone or with anti‐VEGFR2 (ramucirumab) [NCT02597036]. Showed “signs of clinical activity”^h^.
CVX‐241 (Pfizer)	‘CovX‐body’ against VEGF‐A & Ang2	Phase I trial in Advanced Solid Tumors – terminated [NCT01004822]
BI 836880 (Boehringer Ingelheim)	Bispecific Nanobody® against VEGF & Ang2	Phase I trial in Advanced Solid Tumors – Completed [NCT02689505] Active not recruiting [NCT02674152]
RO5520985/ vanucizumab (Roche)	Bivalent IgG1 ‘A2V CrossMab’ against VEGF‐A & Ang2	Phase II trial in Metastatic Colorectal Cancer: with chemotherapy —no PFS benefit (versus bevacizumab+chemotherapy) [NCT02141295]; Phase I trial in Advanced Solid Tumors: with CD40 agonist (RO7009789)—Completed [NCT02665416]—results not yet reported. Phase I trial in Advanced Solid Tumors: +/− PD‐L1 antagonist (atezolizumab)—Completed [NCT01688206]
TAvi6 (Roche)	Tetravalent VEGF‐A & Ang2 Ab	Preclinical^i^
DAAP (G.Y. Koh)	Decoy receptor of VEGF‐A, Ang2, …	Preclinical^j^
CEP‐11981 (Cephalon)	TKI of TIE2, VEGFR1/2/3…	Phase I trial in Advanced Solid Tumors – completed; but further development has ceased^k^
Regorafenib (Bayer)	TKI of TIE2, VEGFR1/2/3, BRAF, RAF1, PDGFRs, FGFRs, KIT, RET, …	As Salvage Therapy for Metastatic Colorectal Cancer: Phase III trial—↑PFS and ↑OS^j^, FDA‐approved. As Adjuvant Therapy for Early Colorectal Cancer: After liver metastasectomy—Terminated [Phase III, NCT01939223]; After postsurgical chemotherapy—Terminated [Phase II, NCT02425683]
Pexmetinib (ARRAY)	TKI of p38 MAPK, TIE2…	Phase I trial in Myelodysplastic Syndromes – completed^l^
Altiratinib (Deciphera)	TKI of MET, TRK, VEGFR2, TIE2, …	Phase I trial in Advanced Solid Tumors – Terminated [NCT02228811]
Rebastinib (Deciphera)	TKI of TIE2, VEGFR2, ABL, …	Phase Ib trial in Advanced Breast Cancer with chemotherapy—recruiting [NCT02824575]

[NCT#], trial identifier on ClinicalTrials.gov database; ITT, ‘intention‐to‐treat’ analysis; mAb, monoclonal antibody; OS, overall survival; PFS, progression‐free survival; TKI, tyrosine kinase inhibitor.

^a^ Monk *et al* (2015).

^b^ Sheridan (2015).

^c^ Al Wadi and Ghatage (2016).

^d^ Monk *et al* (2016).

^e^ Dowlati *et al* (2016).

^f^ Papadopoulos *et al* (2016).

^g^ Scheuer *et al* (2016).

^h^ Martin‐Liberal *et al* (2020).

^i^ Koh *et al* (2010).

^j^ Pili *et al* (2014).

^k^ Grothey *et al* (2013).

^l^ Garcia‐Manero *et al* (2015).

Thus far, unfortunately, Ang/Tie pathway inhibitors have not been successful when compared to VEGF/VEGFR2 inhibitors in a number of phase III clinical trials—at least when tested in late‐stage disease settings where they have been evaluated, as discussed later.

### Molecular biology of Ang‐Tie pathway

**Figure 1:**
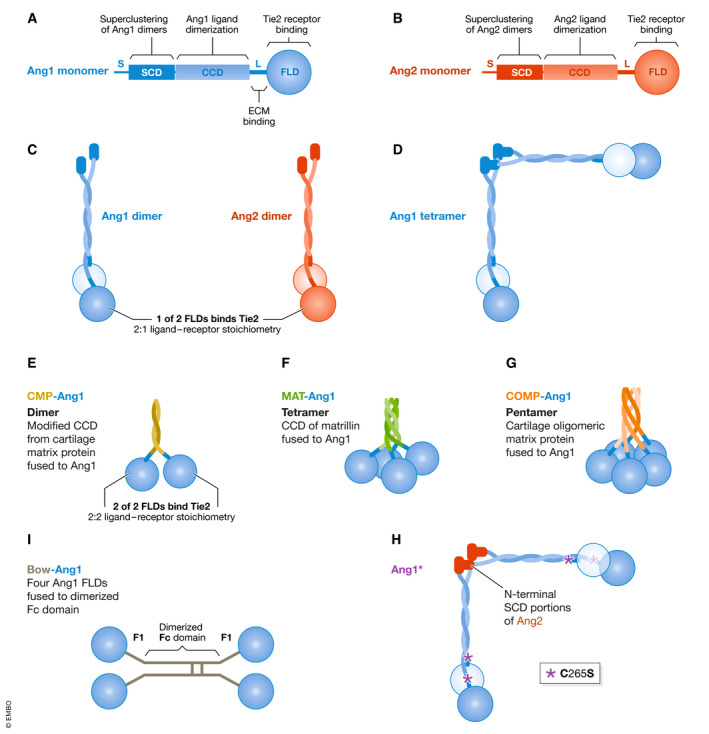
Schematic comparison of Ang1, Ang2, and engineered Ang1 variants/mimetics (A, B) Structural domains of Angiopoietin‐1 (Ang1) and Angiopoietin‐2 (Ang2). FLD = fibrinogen‐like domain. CCD = coiled‐coil domain. SCD = superclustering domain. S = secretory signal. L = linker region. ECM = extracellular matrix. (C) Ang1 and Ang2 monomers form asymmetrical dimers where only one FLD is available to bind Tie2 (the light blue or light red FLD depicts the non‐binding monomer). (D) Ang1 dimers can form tetramers by oligomerization at the SCD, higher‐order oligomers can also be formed (not depicted here) (E) CMP‐Ang1 (modified coiled‐coil domain of cartilage matrix protein fused to Ang1 FLD) to form dimers. (F) MAT‐Ang1 (the coiled‐coil domain of matrillin fused to Ang1 FLD) form tetramers. (G) COMP‐Ang1 (cartilage oligomeric matrix protein fused to Ang1 FLD) form pentamers. (H) Ang1* contains N‐terminal SCD portions of Ang2 and also cysteine‐to‐serine mutations at residue 265 (or residue 245 in the mature protein without the signal peptide). C265S mutation depicted with *. (I) Bow‐Ang1 consists of four Ang1 FLDs fused to a dimerized Fc domain.

**Figure 2:**
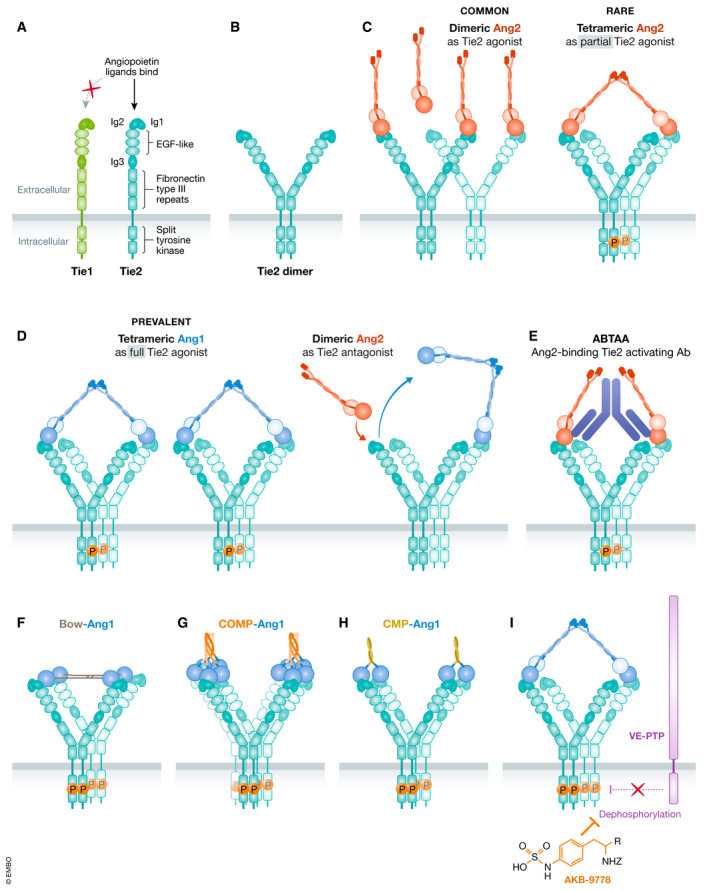
Schematic of Tie2 receptor domains, clustering, and activation (A) Structural domains of monomeric Tie receptors. Angiopoietins bind the 2^nd^ Ig domain (Ig2) of Tie2 (blue) but not of Tie1 (green). (B) In the absence of ligands, Tie2 can form inactive dimers through the membrane‐proximal Fn3 fibronectin type III domain (Leppänen *et al,* 2017; Moore *et al,* 2017). (C) Ang2 exists more commonly in lower‐order oligomeric forms, such as a dimer which does not activate Tie2, and rarely exists in tetramer and higher‐order forms, which can potentially explain its partial agonist activity. (D) Ang1 is the canonical full agonist of Tie2 that predominates in higher‐order forms, such as a tetramer, in which the two FLD’s available for Tie2 binding has been predicted to simultaneously engage two parallel Tie2 dimers within an arrayed complex as depicted (as opposed to the two arms of the same Tie2 dimer) (Leppänen *et al,* 2020). (E) Ang2 competes for the same binding site on Tie2 as Ang1 explaining its antagonistic effect on Ang1‐induced Tie2 phosphorylation. (F) Ang2‐binding Tie2‐activating Antibody (ABTAA) couples together two Ang2 dimers to enable tetrameric engagement and activation of Tie2. (F, G, and H) Tie2 clustering by engineered Ang1 variants/mimetics, Bow‐Ang1‐COMP‐Ang1, and CMP‐Ang1 are confirmed potent agonists of Tie2. As Bow‐Ang1 and COMP‐Ang1 do not contain the native coiled‐coil domains of Ang1, each FLD within the molecule is likely able to bind to a Tie2 ligand‐binding domain. (I) The small‐molecule inhibitor AKB‐9778 inhibits VE‐PTP, a phosphatase of Tie2, hence preserving Tie2 phosphorylation.

Monomeric Ang1 and Ang2 are structurally very similar (Fig. 1A‐B) and share 60% amino acid sequence identity in humans. They both have a C‐terminal fibrinogen‐like domain (FLD) which confers binding to the Tie2 receptor but not to Tie1 (Fig. 2A), a middle coiled‐coil domain (CCD) which facilitates the asymmetrical dimerization of Ang monomers such that only one of the two FLDs is available for Tie2 binding (Fig. 1C), and a shorter N‐terminal region or superclustering domain (SCD) which enables further superclustering of Ang dimers into heterogenous multimeric structures (Fig. 1D) (Davis *et al,* 1996; Maisonpierre *et al,* 1997; Davis *et al,* 2003; Kim *et al,* 2005; Leppänen *et al,* 2020). While dimeric Ang ligands can bind Tie2, this conformation does not induce Tie2 phosphorylation (Fig. 2C) (Davis *et al,* 2003; Leppänen *et al,* 2020). Higher‐order multimeric forms are necessary for activation of intracellular phosphorylation of ligand‐complexed Tie2 receptors (Davis *et al,* 2003). Ang1 is a strong agonist of Tie2 activation whereas Ang2 has context‐dependent roles. Ang2 is an antagonist inhibiting Ang1‐mediated Tie2 phosphorylation but also a weak agonist of Tie2 in the absence of Ang1 (Fig 2C and D).

Kim *et al* demonstrated that when Ang1 molecules are mutated so that they only form dimers or trimers they cannot induce Tie2 phosphorylation *in vitro* (Kim *et al,* 2005). Therefore, it is thought that only higher‐order molecular multimers of Ang1 and Ang2 of tetramer or higher can induce Tie2 phosphorylation (Kim *et al,* 2005). These authors also demonstrated through transmission electron microscopy that the majority of Ang1 molecules exist in trimeric, tetrameric, and pentameric forms bound together by the SCD to form high order molecular forms. However, Ang2 mainly exists in dimer, trimer, and tetramer form, and rarely in high order forms. This is likely a major contributor to the weaker agonistic potential of Ang2 versus the stronger potency of Ang1.

Heparan sulfate glycosaminoglycans (HS‐GAGs) have recently been shown to bind Ang1 and Ang4 resulting in the formation of ternary complexes with Tie2, enhancing Tie2 activation (Griffin *et al,* 2021). HS‐GAGs do not bind Ang2, and this also likely contributes to Ang1 being a more potent activator of Tie2 signaling.

Structural studies have revealed that Tie2 homodimerization is dependent on the transmembrane proximal Fn3 fibronectin type III domain of Tie2 and can exist in a dimer even without bound ligands (Leppänen *et al,* 2017; Moore *et al,* 2017). Similar structural studies also revealed that there is a large 260 Å distance between the ligand‐binding domains of Tie2 homodimers (Fig. 2B; Leppänen *et al,* 2017). Structural analyses of truncated Ang molecules without the N‐terminal SCD have provided novel insights into the ligand conformations necessary for spanning this 260 Å distance and activation of Tie2 receptor complexes (Leppänen *et al,* 2020). Small angle X‐ray scattering (SAXS) experiments revealed that Ang1 and Ang2 likely exist as an extended dimer molecule with the two globular fibrinogen‐like domains (FLDs) from each monomer forming a dimer in an asymmetrical formation (Fig. 1C). Biophysical assays showed that both Ang1 and Ang2 bind to Tie2 in a 2:1 complex formation, where only one FLD of the Ang1 or Ang2 homodimer is able to bind to Tie2 at any given time. Importantly, both Ang1 and Ang2 in dimeric form were unable to induce Tie2 phosphorylation, as was previously shown (Kim *et al,* 2005). The reported limitation in ligand‐to‐receptor stoichiometry is dependent on the coiled‐coil domains, as CMP‐Ang1 dimers (Ang1 with the native coiled‐coil domain replaced with modified dimeric coiled‐coil domain from cartilage matrix protein) can bind to Tie2 in a 2:2 complex formation (Leppänen *et al,* 2020). The authors propose that if these asymmetrical dimers were to further cluster into higher‐order forms by oligomerization of the SCD (as is the case predominantly for Ang1), it is likely that two of the four FLDs in tetrameric Ang would bind diagonally neighboring Tie2 monomers within parallel arrays of dimeric Tie2 receptors (as opposed to the two monomeric arms within a single Tie2 dimer). In this way, Ang FLDs will not bind to the same Tie2 homodimer, but more likely bind to a separate Tie2 receptor (Figure 2C‐D). How this leads to tyrosine kinase autophosphorylation downstream within the array of Tie2 receptors has yet to be elucidated.

Ang2 primarily has Tie2 antagonistic activity by competitively inhibiting binding by oligomeric Ang1 and therefore inhibits Ang1‐mediated clustering and full activation of Tie2. Ang2 further has antagonistic activity via the existence of its smaller splice variant form that lacks a portion of the coiled‐coil domain encoded by exon 2 (Ang2‐_443_), which in turn can be cleaved into a form named Ang2‐_DAP_ (named after the three amino acids at the new N terminus) which lacks the SCD and part of the coiled‐coil domain (Kamiyama & Augustin, 2021; Kapiainen *et al,* 2021). Therefore, Ang2‐_DAP_ is incapable of forming oligomeric complexes and exists mostly in monomeric form, which cannot cause Tie2 activation and in fact acts as a potent Tie2 antagonist by blocking binding of Ang1 or full length and oligomerized Ang2.

Tie1 is mainly expressed by endothelial cells and has been shown to bind to the chemotactic factor leukocyte cell‐derived chemotaxin 2 (LECT2), as well as heparan sulfate glycosaminoglycans (Partanen *et al,* 1992; Xu *et al,* 2019; Griffin *et al,* 2021). Tie1 can be phosphorylated in a Tie2‐dependent fashion when endothelial cells are stimulated with Ang1 (Saharinen *et al,* 2005). Tie1 has roles in regulating Tie2 function in a context‐dependent manner; in sprouting endothelial tip cells, Tie1 negatively regulates the cell surface expression of Tie2, whereas in endothelial stalk cells of blood vessels Tie1 positively maintains Tie2 signaling (Savant *et al,* 2015). During inflammation, proteolytic ectodomain cleavage of Tie1 appears to switch Ang2 from a Tie2 agonist to a Tie2 antagonist, but the exact mechanisms of action remain to be resolved (Kim *et al,* 2016; Korhonen *et al,* 2016).

In addition to differences in multimericity and Tie2 engagement, Ang1 and Ang2 also differ with respect to extracellular matrix binding (Xu & Yu, 2001), as well as effects on Tie1‐Tie2 heterodimers (Seegar *et al,* 2010; D’Amico *et al,* 2014; Savant *et al,* 2015) and activation of integrins (Felcht *et al,* 2012; Lee *et al,* 2013; Hakanpaa *et al,* 2015). A number of thorough reviews of the Ang‐Tie system covering these topics have been published (Thurston *et al,* 2005; Augustin *et al,* 2009; Huang *et al,* 2010; Thurston & Daly, 2012; Gerald *et al,* 2013; Koh, 2013; Kiss & Saharinen, 2017; Saharinen *et al,* 2017).

### *Clinical* *development of Anti‐Ang2 agents*

**Figure 3:**
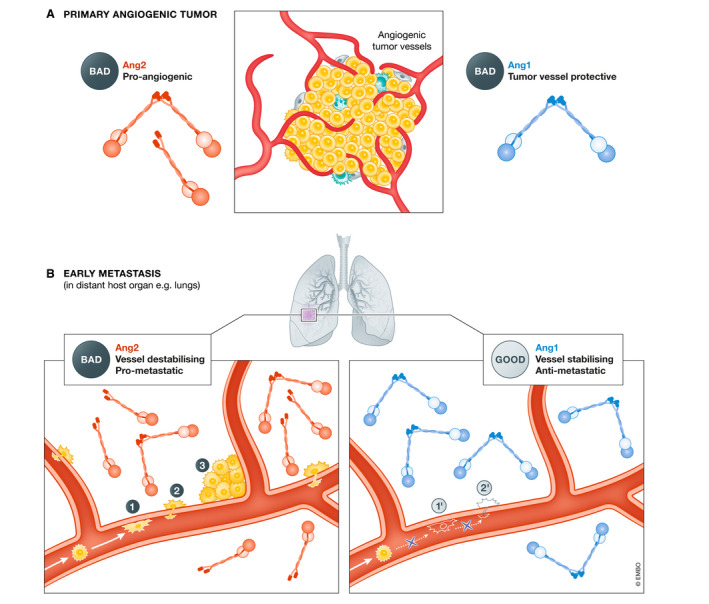
Differential roles of Ang1 and Ang2 in established growing tumors vs. metastatic dissemination (A) At the site of established growing tumors, Ang2 acts as an angiogenesis initiator, while exogenous Ang1 diminishes the anti‐tumor efficacy of anti‐angiogenic anti‐VEGF and anti‐Ang2 therapies (Falcón *et al,* 2009; Huang *et al,* 2009; Coxon *et al,* 2010; Daly *et al,* 2013). (B) At the destination sites of metastasis, Ang2 de‐stabilizes the "normal" host blood vessels to facilitate various steps in the metastatic cascade (Talmadge & Fidler, 2010). (1) Tumor cell adhesion to capillary wall (Kim *et al,* 2001); (2) tumor cell extravasation (Gavard *et al,* 2008); and (3) tumor cell co‐option of existing host vessels (Holash *et al,* 1999). We thus hypothesize that Ang1, a vascular stabilizing factor, may inhibit these early metastatic events, i.e., in the lung: (1') inhibition of cancer cell adhesion and arrest (Michael *et al,* 2017); and (2') inhibition of extravasation due to stabilized vessels (Wu *et al,* 2015).

Amgen Inc. was the first to introduce Ang2/Ang1‐dual blockers into clinical testing with the dual targeting “peptibody”, AMG386/trebananib. This drug struggled in three phase III advanced ovarian cancer trials—showing no OS benefit and no consistent PFS benefit in the second‐line treatment setting in the TRINOVA‐1 and TRINOVA‐2 clinical trials (Monk *et al,* 2014, 2015) (except in a subgroup of TRINOVA‐1 patients with ascites at baseline, in which case there was an OS of 14.5 vs. 12.3 months, paclitaxel plus trebananib vs. placebo, respectively) (Monk *et al,* 2016). In the first‐line treatment setting in the TRINOVA‐3 clinical trial, there were no PFS benefits and no global OS benefit (Al Wadi & Ghatage, 2016). All three trials involved combinations of trebananib with chemotherapy (Sheridan, 2015). Trebananib also failed to prolong PFS in phase II trials involving metastatic RCC (Rini *et al,* 2012), metastatic CRC (Peeters *et al,* 2013), metastatic gastro‐esophageal cancer (Eatock *et al,* 2013), and metastatic breast cancer (Diéras *et al,* 2015). Recently published phase II trials combining bevacizumab and trebananib in patients with recurrent glioblastoma or gliosarcoma indicated that the addition of trebananib to bevacizumab actually shortened PFS (Lee *et al,* 2020). Additionally, development of Amgen’s bispecific Ang1/Ang2 antibody, AMG780, was halted at the phase I clinical trial level (Table 2). The original rationale for using Ang2 inhibitors with concurrent Ang1 inhibition has been called into question (Sheridan, 2015). The original premise, backed by some preclinical primary tumor therapy studies, was that Ang1 might act as a maturation or ‘normalizing’ (stabilizing) factor for tumor blood vessels (Falcón *et al,* 2009; Coxon *et al,* 2010). Consequently, excess Ang1 may dampen the efficacy of anti‐angiogenic anti‐VEGF or anti‐Ang2 therapies (Huang *et al,* 2009; Daly *et al,* 2013); and that concurrent Ang1 inhibition might additionally allow for the regression of existing tumor blood vessels while Ang2 inhibition prevents the sprouting of new tumor blood vessels (Falcón *et al,* 2009; Coxon *et al,* 2010). These speculations may be valid when conceptualizing the role of Ang1 on angiogenic tumor blood vessels (Fig. 3A). On the other hand, when considering the role of Ang1 as a stabilizer of quiescent normal blood vessels systemically, Ang1 inhibition can be a “double‐edged sword” (Cascone & Heymach, 2012). Ang1 inhibition may also detrimentally remove an endogenous gatekeeper of endothelial‐barrier function, which would be normally expected to impede metastatic cell dissemination (extravasation) to distant host organs (Fig. 3B). This possibility has been reinforced by findings involving Ang1 genetically deficient mice; while there were no observed effects on transplanted primary tumor growth, lung metastatic burden was increased in these animals in comparison with wild‐type mice (Michael *et al,* 2017).

In contrast, several Ang2‐monospecific antibodies are still in active clinical development, including REGN910 (nesvacumab) from Regeneron, MEDI‐3617 from MedImmune/AstraZeneca (while discontinued for monotherapy use (Hyman *et al,* 2018) it is currently being evaluated in combination with a CTLA‐4 checkpoint inhibitor in a phase I clinical study), as well as the monoclonal Ang2 antibody LY3127804 from Eli Lilly (Table 2). Similarly, the VEGF‐A/Ang2‐bispecific antibody targeting agents, BI836880 from Boehringer Ingelheim and vanucizumab from Roche, remain under clinical investigation. The latter antibody has been evaluated in combination with PD‐L1 blocking antibodies or stimulatory agonist antibodies directed to CD40 immune checkpoint molecules in phase I clinical studies (Table 2).

### *Clinical* *development of Tie2 inhibitors*

There are several small‐molecule tyrosine kinase inhibitor (TKI) drugs that inhibit Tie2 (Table 2), many of which also inhibit VEGFR2. Regorafenib has progressed through phase III clinical trial evaluation and achieved regulatory approval as a salvage therapy for metastatic CRC (Grothey *et al,* 2013). Ripretinib, a TKI that was primarily designed to inhibit c‐kit and PDGFRα but also inhibits Tie2 and VEGFR2 (Smith *et al,* 2019), has been approved for use as a salvage therapy for mutant c‐kit expressing gastrointestinal stromal tumors (Dhillon, 2020). Given that these TKIs have multiple targets and are not restricted to a single receptor tyrosine kinase, it is difficult to determine the specific contribution of Tie2 inhibition to the efficacy of regorafenib or ripretinib. It has been suggested that Tie2 inhibitors may not actually be superior to Ang2 inhibitors. A preclinical study comparing an anti‐Ang2 antibody (REGN910) with an antagonistic anti‐Tie2 antibody (REGN1376) reported similar efficacy in six out of seven tumor models evaluated (Adler *et al,* 2014). This anti‐Tie2 antibody blocks both Ang1 and Ang2 binding to Tie2, implying that concurrent Ang1 inhibition may not necessarily boost the efficacy of Ang2 inhibition. This result is in agreement with another preclinical study that did not find increased activity by directly adding an Ang1 inhibitor to an Ang2 inhibitor (Wang *et al,* 2014).

## The rationale for and design of novel “Pro‐Ang1” therapeutic strategies

The literature summarized above suggests that co‐inhibition of Ang1 and Ang2 may not be an effective approach for treating metastatic disease or restraining early metastatic spread. Taking this a step further, it is important to examine the question whether Ang1 supplementation or Tie2 activation can be an effective anti‐metastatic strategy in the pre‐ or postoperative treatment setting. More specifically, can the systemic administration of Ang1 analogues or Tie2 activators sufficiently stabilize the normal blood vessels of distant host organs to suppress early metastatic events—e.g., capillary adhesion of circulating tumor cells, transendothelial migration of tumor cells and co‐option of existing host blood vessels by extravasated tumor cells? While Ang1 mimetic supplementation and alternative Tie2 activators both have the same ultimate outcome of activating Tie2, they work in different ways and may have somewhat different effects on other components of the Ang/Tie pathway including integrin binding. For these reasons, we discuss them as separate distinct strategies.

To explore these questions, several distinct approaches have been undertaken in preclinical studies. Our group assessed the use of direct Ang1 supplementation therapy—using either a putative Ang1 mimetic peptide drug candidate called Vasculotide (Wu *et al,* 2015), or alternatively, well‐characterized engineered variants of Ang1 called Bow‐Ang1 and COMP‐Ang1 (Wu *et al,* 2016b). As we could not confirm whether Vasculotide binds to or activates Tie2 (Wu *et al,* 2015), we will not discuss this agent further and will address the relevance of the other agents.

### *Engineered* *variants or mimetics of Ang1 that activate Tie2*

Various versions of Ang1 have been engineered to improve solubility, stability, and uniformity compared with recombinant native Ang1. For instance, Koh and colleagues have developed a series of reagents that oligomerizes the native FLD of Ang1 using shorter alternative CCDs—e.g., using the CCD of cartilage oligomeric matrix protein (COMP) to make pentameric “COMP‐Ang1” (Cho *et al,* 2004a), the CCD of matrilin 1 (MAT) to make tetrameric “MAT‐Ang1” (Cho *et al,* 2004a), or the CCD from cartilage matrix protein (CMP) with site‐directed mutagenesis to create a dimeric but nonetheless potent “CMP‐Ang1” (Oh *et al,* 2015) (Fig. 1E–G). Regeneron developed “Ang1*” by replacing the N‐terminal domain of Ang1 with the N‐terminal domain of Ang2 and a cysteine‐to‐serine mutation at residue 265 (residue 245 of the mature signal peptide cleaved protein) (Davis *et al,* 2003) (Fig. 1H). Regeneron also developed “Bow‐Ang1” (or Ang1‐Fd‐Fc‐Fd or Ang‐F1‐Fc‐F1, where Fd or F1 is an Ang1 FLD) by fusing four native FLD’s of Ang1 to dimerized Fc domains from IgG1 (Davis *et al,* 2003) (Fig. 1I). These reagents are well‐characterized and validated in terms of molecular structure, Tie2‐binding affinity, and Tie2 agonistic activity (Table 3). These modified Ang1 variants all utilize the native FLD of Ang1 to bind and cluster Tie2 receptors to induce Tie2 phosphorylation (Fig. 2F, G and H). COMP‐Ang1 is a potent Tie2 agonist despite the likelihood of it being unable to span the Tie2 homodimer gap (Leppänen *et al,* 2017). The increased capacity of COMP‐Ang1 to activate Tie2 when compared with endogenous Ang1 can potentially be explained by its lack of native Ang1 CCD (as discussed earlier for CMP‐Ang1) allowing each FLD in the pentamer to cluster together multiple Tie2 molecules in *cis* on the same cell surface or in *trans* juxtapositionally between EC‐EC junctions (Fig 2G).

**Table 3:** Comparison of Tie2 activators: theoretical mechanisms & experimental evidence.

Agent	Theoretical Mechanism of Tie2 Activation	Molecular Structure	Target Binding	Tie2 Agonism (induction of Tie2 phosphorylation)	Target Specificity
Recombinant Ang1 variants					
COMP‐Ang1	Recombinantly oligomerizing the globular Tie2‐binding domain (FLD) of native Ang1. By binding and clustering Tie2, triggers Tie2 phosphorylation.	Confirmed pentamers ~175‐200 kDa [SDS–PAGE; TEM]^a^ [SEC]^b^	Strong binding to Tie2: K_D_ ≈ 3× Ang1’s [PD;SPR]^a^; K_D_ ≈ 6× Ang1’s [SPR]^a^	More potent than Ang1 [IPIB‐HUVEC^a^; IPIB‐lung^c^]	Does not bind Tie1 [PD]^a^, ^d^
Bow‐Ang1		Confirmed tetramers ~147.5 kDa [MALS]^e^	Strong binding to Tie2: IC_50_ ≈ 6× Ang1’s [SPR]^e^; K_D_ ≈ 3× Ang1’s [SPR]^d^ Clusters Tie2 via 1:1 binding stoichiometry between FLD and Tie2 [SPR]^e^	Comparable to Ang1 [IPIB‐EA.hy926]^e^	N/A
Small‐molecule VE‐PTP inhibitor					
AKB‐9778	By inhibiting VE‐PTP (or HPTPβ), a protein tyrosine phosphatase that dephosphorylates Tie2.	Confirmed structural orientation of phosphotyrosine‐mimetic component [XRC]^g^	Confirmed binding to VE‐PTP [XRC]^f^	Comparable to Ang1 when given alone [IPIB‐HUVEC; IPIB‐retina]^g^ Strongly enhances Ang1 and Ang2‐induced Tie2 phosphorylation [IPIB‐HUVEC]^g^	Selective inhibitor of HPTP‐β/η/μ versus other PTPs [CEI]^g^
Antibody					
ABTAA (Ang2‐binding Tie2‐activating antibody)	By binding and clustering Ang2, turns Ang2 into Ang1‐like oligomeric ligands that cluster and activate Tie2.	~160‐kDa [SEC; SDS–PAGE]^h^	Strong binding to human Ang2 (K_D_=0.2nM) & cross‐reactive to mouse Ang2 (K_D_=138nM) [SPR]^h^ without inhibiting Ang2‐Tie2‐binding [cELISA]^h^ Clusters Tie2 via 1:2 binding stoichiometry between ABTAA & Ang2.FLD‐Tie2.ECD [MALS]^h^	Strongly induce Tie2 phosphorylation in the presence of Ang2 [IPIB‐HUVEC; IPIB‐HMVEC_L_; IPIB‐lung]^h^ & subsequent Tie2 internalization/ endocytosis [IF]^h^	Does not bind Ang1 [cELISA]^h^
AB‐Tie1‐39 (Tie1 binding human antibody)	Unknown	N/A	High affinity binding to human and mouse Tie1^i^	Induces Tie2 phosphorylation *in vivo* in lung endothelium, but inhibits Tie2 phosphorylation *in vitro* ^i^	Immunoprecipiates Tie1 but not Tie2^i^

cELISA, competitive ELISA; CEREP, commercial high‐throughput binding assays; ‐EA.hy926, using cultured hybrid endothelial‐tumor cell lysates; ‐HMVEC_L_, using cultured primary human lung microvascular endothelial cell lysates; –HUVEC, using cultured primary human umbilical venous endothelial cell lysates; IF, immunofluorescent staining of cultured ECs; IPIB, immunoprecipitating for Tie2 before immunoblotting for phospho‐tyrosines; LS, light scattering analysis of molecular weight; ‐lung, using homogenates of *in vivo*‐treated mouse lungs; MALS, multi‐angle light scattering; N/A, not available; PD, pull‐down of purified ligands using sTie2‐Fc or sTie1‐Fc coupled to protein A agarose beads; SDS–PAGE, molecular weight analysis by sodium dodecyl sulfate‐polyacrylamide gel electrophoresis, under non‐reducing vs. reducing conditions; SEC, size‐exclusion chromatography by FPLC; SPR, surface plasmon resonance by BIAcore; TEM, visualization by transmission electron microscopy; XRC, X‐ray crystallography.

^a^ Cho *et al* (2004a).

^b^ Oh *et al* (2015).

^c^ Cho *et al* (2004b).

^d^ Wu *et al* (2015).

^e^ Davis *et al* (2003).

^f^ Amarasinghe *et al* (2006).

^g^ Shen *et al* (2014).

^h^ Han *et al* (2016).

^i^ Singhal *et al* (2020).

### *Other* *novel Tie2 activators*

**Figure 4:**
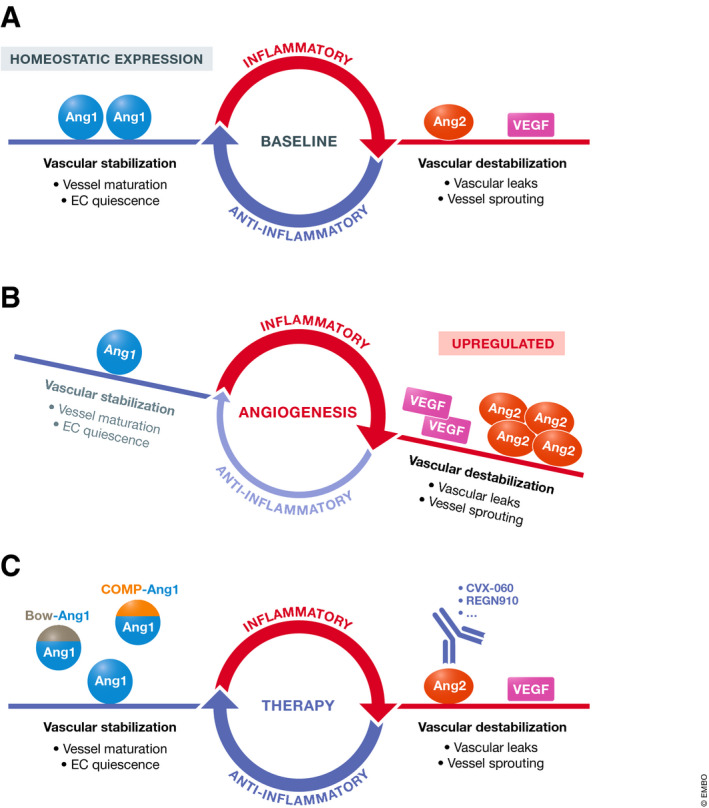
Targeting the Angiopoietins in Cancer Therapy (A) Ang1 is constitutively expressed in low levels in healthy adults and maintains endothelial quiescence (Pfaff *et al,* 2006). (B) In many cancer types, overexpression of Ang2 and increased Ang2/Ang1 ratios correlate with more advanced disease stages and worse prognosis (Huang *et al,* 2010). (C) There are Ang2‐neutralizing agents already in clinical development as oncologic drugs, as well as preclinical investigatory agents activating Tie2.

Several novel Tie2 activators have been described, none of which involves modification or mimicry of Ang1 (Table 3). The first report of a Tie2‐activating antibody was described in 2001, whereby a monoclonal antibody to human Tie2 was generated that could induce Tie2 autophosphorylation in HUVEC (Hansbury *et al,* 2001). ABTAA (Ang2‐binding Tie2‐activating antibody) is a unique humanized monoclonal IgG1 antibody (Han *et al,* 2016). It clusters Ang2 ligands into higher‐order oligomers likely allowing Ang2 to span the gap between Tie2 ligand‐binding domains, inducing clustering of Tie2 and activating downstream signaling in an Ang1‐like manner (Fig. 2E; Han *et al,* 2016; Leppänen *et al,* 2017). AKB‐9778 is a small‐molecule inhibitor with high specificity for the protein tyrosine phosphatase known as vascular endothelial protein tyrosine phosphatase (VE‐PTP) in mice (or HPTP‐β in humans), which is responsible for dephosphorylating Tie2 during negative feedback regulation (Goel *et al,* 2013; Shen *et al,* 2014; Fig. 2I). AB‐Tie1‐39 is a recently described Tie1 binding antibody which has been shown to cause phosphorylation of Tie2 in lung endothelium *in vivo* and inhibition of cancer cell extravasation (Khan & Kerbel, 2020; Singhal *et al,* 2020). The Tie2‐activating mechanism of this antibody is unknown, and in fact counterintuitively, this antibody was originally designed to bind to Tie1 and inhibit Tie2 phosphorylation *in vitro*, adding to the complexity of the mechanism of action. These aforementioned Ang2 inhibitors and Tie2 activators have the potential to stabilize the vasculature, reduce vessel leaks, and reduce metastasis formation (Fig. 4).

## Insights from studying preclinical modeling of therapy of early‐stage metastatic disease

Approximately 90% of all cancer‐related deaths are due to metastatic disease (Anderson *et al,* 2019). The metastatic cascade is complex but basically involves tumor cells invading their local microenvironment, intravasation of tumor cells into the bloodstream, survival in the blood, and eventual arrival at a distant site where tumor cells extravasate and begin to grow at the new site, or in some cases lie dormant (Anderson *et al,* 2019; Hapach *et al,* 2019). Early (stage) metastasis is defined as an absence of overt metastases, as assessed by imaging, but mice or patients may nevertheless be at risk of developing overt metastatic disease from occult micrometastatic lesions or even single cells. However, the field of preclinical cancer therapy research is still dominated by short‐term localized (non‐metastatic) primary tumor therapy studies, whether using transplanted tumor cell lines, genetically engineered mouse models (GEMMs) or human patient‐derived xenografts (PDXs). Indeed, most GEMMs or PDX models are poorly or non‐metastatic (Francia *et al,* 2011). As we have advocated before, the use of preclinical models that simulate different stages of metastatic disease (i.e., early and late stages) is paramount when developing and testing new cancer therapeutics, to avoid restricting studies solely to the evaluation of effects on established primary tumor growth (Ebos & Kerbel, 2011; Francia *et al,* 2011). More preclinical studies should be undertaken to recapitulate a common clinical scenario, i.e., surgical resection of primary tumors with curative intent, which then allows modeling of perioperative treatment settings such as neoadjuvant (Ebos *et al,* 2014) and adjuvant systemic therapies (Ebos & Kerbel, 2011). These models additionally recapitulate how the presence of a primary tumor can aid in the formation of a pre‐metastatic niche (Doglioni *et al,* 2019), and how surgical resection itself can induce systemic immunosuppression which may also aid in growth of metastases or dormant tumor cells (Krall *et al,* 2018).

### *Combining* *Anti‐Ang2 Agents with VEGF/VEGFR2 inhibitors*

With that perspective in mind, we performed a study (Wu *et al,* 2016a), where orthotopic primary tumors in mice were resected to explicitly model the response of postsurgical micrometastatic disease to systemic adjuvant therapies. Two different approaches to dual targeting of the Ang2/Tie2 and VEGF/VEGFR2 pathways were examined. The first involved the combination of an Ang2‐specific modified antibody (CVX‐060) and a small‐molecule VEGFR2 TKI (such as sunitinib or regorafenib). The second approach utilized a single agent, CVX‐241, a modified antibody that is bispecific for Ang2 and VEGF‐A. Interestingly, the first approach was more effective than the second approach in a kidney cancer model, but the reverse was true in breast cancer models. In the resected orthotopic MDA‐MB‐231.LM2‐4 model, which is an aggressive metastatic variant of MDA‐MB‐231 (a triple‐negative human breast cancer cell line), adjuvant CVX‐241 therapy prolonged median OS while adjuvant CVX‐060 plus sunitinib did not. In the resected orthotopic EMT6/CDDP murine breast cancer model, adjuvant CVX‐241 therapy also showed a trend of increasing median OS—performing better than a PD‐L1 immune checkpoint inhibitor. Further, in the resected orthotopic RENCA murine renal carcinoma model, however, adjuvant CVX‐060+sunitinib therapy was superior to adjuvant CVX‐241 therapy.

### *Combining* *Ang2/VEGF co‐targeting with metronomic chemotherapy*

Another one of the few studies that has examined anti‐Ang2 therapy with respect to metastatic disease explicitly modeled adjuvant therapy for postsurgical micrometastatic disease (Srivastava *et al,* 2014). This study reported substantial efficacy when combining a different anti‐Ang2 antibody (LC06 clone derivate) with low‐dose metronomic (LDM) chemotherapy in the adjuvant therapy setting. In their resected orthotopic 4T1 mouse breast cancer model, the combination of adjuvant LDM paclitaxel plus anti‐Ang2 therapy led to a greater inhibition of postsurgical metastases and greater prolongation of OS than either therapy alone, although anti‐Ang2 monotherapy still resulted in survival advantages in comparison with control IgG. In their subcutaneous (ectopic) Lewis lung carcinoma (LLC) resection model, the triple combination of adjuvant LDM gemcitabine chemotherapy, the Ang2 antibody, and a VEGF antibody led to maximal inhibition of postsurgical lung metastases. The authors attributed the therapeutic anti‐metastatic effects to both the anti‐angiogenic effect of Ang2 blockade and blocking of Ang2‐mediated upregulation of adhesion molecules and chemokines leading to infiltration of tumors by pro‐metastatic macrophages.

### *Anti‐Ang2* *agents as preferential inhibitors of micrometastatic disease*

**Figure 5:**
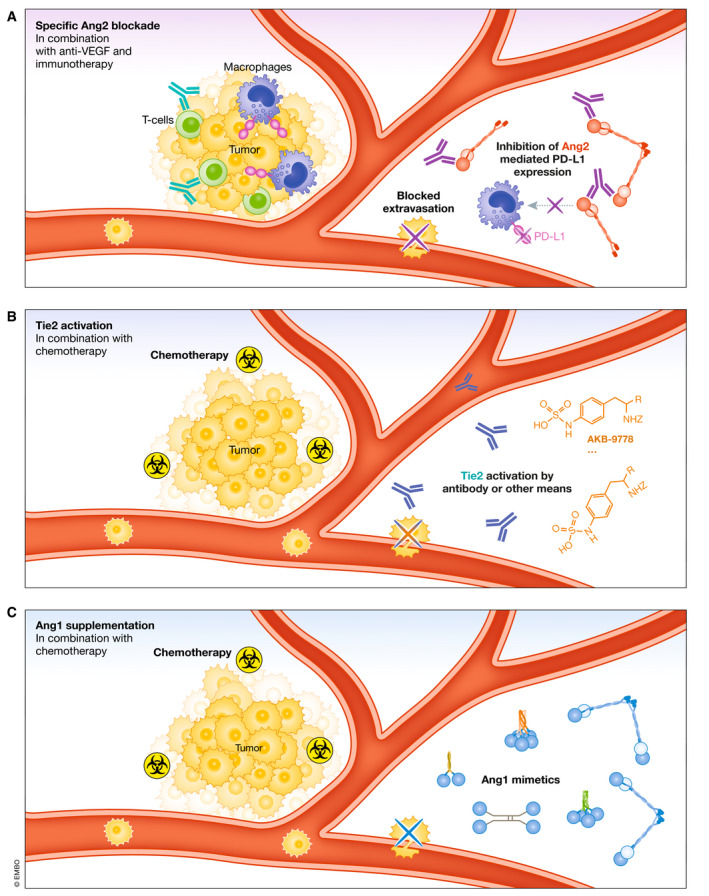
Potential strategies for use of Ang/Tie targeting agents in combination with other therapies for inhibiting and/or treating early‐stage metastatic disease in, e.g., the lung Tumor cells represent a micrometastatic lesion in a distant organ such as the lung. (A) Specific Ang2 blockade, along with its anti‐metastasis and vascular effects, blocks Ang2‐mediated upregulation of PD‐L1 on M2 macrophages. Ang2 blockade has the potential to reduce the immunosuppressive effects of PD‐L1 in the tumor microenvironment, which could be an ideal combination for immunotherapies such as agonist CD40 antibodies or PD‐1 blocking antibodies (green). Ang2 blockade also strengthens endothelial cell‐endothelial cell junctions inhibiting extravasation of tumor cells, along with a multitude of other effects described in the main text. Combining with VEGF blockade would also likely enhance the effects on the vasculature and relieving of immunosuppression. (B) Tie2 activation, whether induced by antibody drugs (AB‐Tie1‐39, ABTAA) or maintained by a small‐molecule inhibitor (AKB‐9778), potentially modulates organ vessels at possible sites of metastasis to reduce tumor cell extravasation and vessel co‐option. (C) Ang1 supplementation, in addition to activating Tie2, may additionally involve crosstalk with integrins to modulate vessels at metastatic sites and inhibit tumor cell extravasation. Moreover, both Ang1 supplementation and Tie2 activation may enhance the intratumoral delivery of chemotherapy, could be partnered with immunotherapy, and treat already seeded micrometastases, although this has not been described experimentally.

Both Wu *et al,* 2016 and Srivastava *et al,* 2014 suggest that certain postoperative treatment combinations involving anti‐Ang2 agents may be worthy of future clinical trial evaluation (Srivastava *et al,* 2014; Wu *et al,* 2016a). Additionally, a recent study reported perioperative neoadjuvant plus adjuvant treatment of trebananib in an ectopic PDX model of renal cell carcinoma (Elbanna *et al,* 2020). In this case, mice were treated with trebananib before resection of subcutaneous primary tumors and treatment was continued post‐surgery; this strategy resulted in prolonged survival and reduced lung metastasis and both effects were enhanced when the drug was combined with a MET‐targeted TKI. These results in early disease are in contrast to recent failures of trebananib (once the leading investigational agent targeting Ang/Tie pathway) in phase II/III clinical trials involving late‐stage metastatic cancers, as discussed earlier. Investigational drugs are virtually never tested in long‐term expensive adjuvant therapy clinical trials unless they are already shown to be efficacious in the advanced metastatic setting and any exceptions usually have to be supported by compelling biological rationale and preclinical evidence such as those targeting dormancy (Mina & Sledge, 2011; Ganesh & Massagué, 2021). We would argue, however, that there is strong rationale to consider anti‐Ang2 therapeutics as candidate ‘preferential inhibitors of early metastatic disease’ (Fig. 5A). There are several lines of evidence to support this approach. First of all, in a preclinical study of subcutaneous primary tumors, Ang2 was pro‐tumorigenic during early phases of tumor growth but relatively dispensable in later stages (Nasarre *et al,* 2009). Conceivably, Ang2 could similarly have a greater role in promoting growth of micrometastases compared with macrometastases (Sheridan, 2015), and Ang2 blockade may yield equally or more potent inhibitory effects on micrometastases compared with its inhibitory effects observed on established metastatic lesions (Mazzieri *et al,* 2011). Secondly, Ang2 engages different signaling pathways in quiescent ECs versus angiogenic ECs. In resting blood vessels, Ang2 acts primarily as a vessel‐destabilizing factor through Tie2 antagonism (Felcht *et al,* 2012). During sprouting angiogenesis, tip ECs decrease their Tie2 expression, yet Ang2 can still regulate EC migration via integrin signaling (Felcht *et al,* 2012). Conceivably, anti‐Ang2 therapeutics could have differential efficacy when used in the adjuvant setting (to target pre‐existing quiescent host blood vessels) versus in advanced metastatic disease (to target angiogenic tumor blood vessels). Thirdly, the early growth of small tumors is potentially sustained by co‐opting the existing blood vessels of highly vascularized host organs, with strong induction of Ang2 in these co‐opted ECs (Holash *et al,* 1999; Kuczynski *et al,* 2019). Thus, in the postsurgical setting, Ang2 could potentially be an earlier and more relevant target than VEGF, when small micrometastases are not yet heavily dependent on VEGF‐driven neo‐angiogenesis.

With respect to therapy of metastatic disease, Ang2 blockade and genetic deletion has been studied mostly in the context of lung metastasis; however, a study investigating Ang2 KO mice and the ability of MC38 colon cancer cells to form lung or liver metastases found that lack of Ang2 protected mice from lung metastases but actually enhanced liver metastases (Im *et al,* 2013). Although, whether this proves to be the case for therapeutic Ang2 blockade and not global genetic Ang2 deletion remains to be investigated. Such differences could be attributed to molecular mechanisms uncovered in lung endothelial cells and the formation of the pre‐metastatic niche which may not be the case for liver endothelial cells (Minami *et al,* 2013; Rigamonti & De Palma, 2013). VEGF secreted from tumor cells activates NFAT signaling in lung endothelial cells which induces expression of Ang2 locally (Minami *et al,* 2013), which in turn causes weakening of endothelial cell junctions allowing extravasation of tumor cells and facilitating formation of metastases (Holopainen *et al,* 2012). It is possible that the effects noted in lung tissue could also extend to lymph nodes. This is supported by evidence showing that adenoviral‐mediated overexpression of Ang2 can mediate increased lung and lymph node metastases (Holopainen *et al,* 2012). A recent study has implicated Ang2 as being critical for metastatic dissemination of tumor cells into the lymphatic vasculature (Gengenbacher *et al,* 2020). This study utilized models of melanoma involving implanted tumor fragments from GEMMs which maintained lymphatic vessels within primary tumors, this allowed metastatic dissemination in the lymphatic vasculature, whereas cell line implanted tumors did not. When Ang2 was blocked in a neoadjuvant treatment setting before surgical resection in these models, this led to greatly enhanced survival. Conversely, when Ang2 was blocked in an adjuvant treatment setting after surgical resection there were no significant differences in OS. The authors then went on to show that Ang2‐Tie2 signaling is critical for maintenance of intratumoral lymphatic vessels and upon Ang2 blockade these vessels are diminished along with their potential to seed distant metastases. Ang2 blockade may be a critical neoadjuvant therapeutic target in tumor types that seed lymphatic metastases but do not yet display lymph node metastases.

### *Ang2‐selective* *vs. Ang2/Ang1‐dual inhibition?*

As mentioned previously, REGN910 (an anti‐Ang2 antibody) was reported to be as efficacious as REGN1376 (an anti‐Tie2 antibody, that blocks both Ang1 and Ang2 binding) in six out of seven preclinical tumor models (Adler *et al,* 2014). In a separate study, REGN1376 also did not show greater efficacy than REGN910 as an add‐on to adjuvant chemotherapy in a model of resected orthotopic primary triple‐negative breast cancer (TNBC) (Wu *et al,* 2016b). One implication of these findings could be that among the anti‐Ang2 agents currently under clinical development, some of the emerging candidates—the Ang2‐monospecific inhibitors (e.g., nesvacumab) and Ang2/VEGF‐bispecific inhibitors (e.g., vanucizumab)—might hold more promise than the earliest candidate, trebananib, which was an Ang2/Ang1‐dual targeting inhibitor, but especially in the perioperative treatment setting. Another study compared two antibody clones, one which displayed higher selective inhibition of Ang2 over Ang1 (clone LC06) and one that similarly inhibited both Ang1 and Ang2 (clone LC08) (Thomas *et al,* 2013). While both antibodies inhibited primary tumor growth and lung metastasis to a similar degree, blockade of both Ang1 and Ang2 led to the regression of healthy vessels in the mouse trachea, whereas Ang2 blockade alone did not. Therefore, selective Ang2 inhibition may also have less off‐target side effects on healthy vessels compared with dual blockade.

### *Perioperative* *use of "Vascular Stabilizers": Ang1 supplementation*

Using the Bow‐Ang1 and COMP‐Ang1 agents to activate Tie2, we previously conducted a study that was the first to directly simulate perioperative Ang1 supplementation therapy in a preclinical model of resectable breast cancer, following orthotopic implantation of a human TNBC cell line (MDA‐MB‐231.LM2‐4) in SCID mice (Wu *et al,* 2016b). Prior published studies involved only testing Ang1 supplementation on unresected primary tumors—a setting in which Ang1 supplementation actually diminished the activity of co‐administered anti‐Ang2 (Daly *et al,* 2013) or anti‐VEGF (Huang *et al,* 2009) therapy. In this study that incorporated surgical resections of primary tumors, the addition of short‐term perioperative Ang1 supplementation (1 day preoperative + 9 days postoperative) improved the OS benefit of adjuvant paclitaxel chemotherapy for TNBC, especially when further combined with adjuvant anti‐VEGF therapy via the VEGF trap drug, aflibercept (Wu *et al,* 2016b) (Table 4). The preoperative/neoadjuvant phase of Ang1 supplementation was intentionally kept short to minimize the risks of exposing primary tumor blood vessels to exogenous Ang1 and to maximize the treatment of blood vessels at potential metastatic sites during the postoperative/adjuvant phase (see Fig. 3). These beneficial effects could also be partly explained by Ang1‐mediated enhanced intratumoral drug delivery of chemotherapy, as Ang1 supplementation alone failed to increase OS (Wu *et al,* 2016b). In previous studies, Ang1‐COMP was shown to increase the delivery of 5‐fluorouracil chemotherapy into primary LLC tumors (Hwang *et al,* 2009) and Tie2 activation has also been shown to increase drug delivery into tumors (Park *et al,* 2017).

**Table 4:** Effects of Tie2 activators in preclinical cancer studies.

Agent	Effects on Host	Effects on Primary Tumors	Effects on Metastases
COMP‐Ang1	[+] ↓ Host organ damage from ionizing radiation: by reducing *intestinal* EC apoptosis^[P]^ ^a^; protecting ECs and hematopoietic cells in *bone marrow* to ↓ myelosuppression^[^ ^a^ ^]^	[○] Monotherapy does not ↑ tumor growth in s.c. LLC model ^[^ ^a^ ^]^ ^c^ [+] In combination, ↑ anti‐tumor activity of chemotherapy on s.c. LLC tumors by “normalizing” tumor blood vessels & ↑drug delivery ^[^ ^c^ ^]^ ^c^ [−/+] Slight trends of ↑growth but ↓invasiveness of orthotopic LM2‐4 tumors^[P]^ ^d^	[−] Treatment of primary s.c. LNM35 tumors ↑ metastatic dissemination to the lungs—via blood vessel enlargement and increased tumor cell intravasation and extravasation^[^ ^d^ ^]^ ^e^ [○] Concurrent perioperative use does not improve adjuvant sunitinib therapy in resected orthotopic LM2‐4 model^[P]^ ^d^
Bow‐Ang1	Not examined.	[○/−] Monotherapy did not promote tumor growth, but concurrent use reversed inhibitory effects of Ang2 blocker on tumor growth/angiogenesis in s.c. Colo205/A431 models^f^ [−] Long‐term use reduced tumor responsiveness to VEGF blockade by stabilizing tumor blood vessels in orthotopic SK‐NEP‐1 model^g^ *(*Huang *et al,* 2009 *)* [−/+] Slight trends of ↑growth but ↓invasiveness of LM2‐4 tumors^d^	[+] Perioperative short‐term use, +/−aflibercept, ↑OS of adjuvant paclitaxel chemotherapy in resected orthotopic LM2‐4 model^d^
AKB‐9778	[○] ↓Permeability & ↑diameter of blood vessels in skin^i^ [○] No change in systemic blood pressure^i^	[○] Monotherapy does not accelerate primary tumor growth in orthotopic 4T1, E0771, P0008, MMTV‐PyVT breast cancer models^i^ [+] Stabilized blood vessels, ↑perfusion, ↓hypoxia, and ↑radiation response in established orthotopic 4T1 primary tumors *(*Goel *et al,* 2013 *)*	[+] Adjuvant monotherapy ↓ lung metastases in resected orthotopic 4T1 model by inhibiting tumor cell extravasation^i^ [+] Addition to adjuvant doxorubicin chemotherapy showed trend of ↑OS in resected orthotopic 4T1 model *(*Goel *et al,* 2013 *)*
ABTAA	[○] No vascular changes in kidneys, cornea, tracheal mucosa, and ear skin of normal mice^j^	[+] ABTAA normalized tumor vessels, ↓tumor hypoxia, favorably altered TAMs and T_reg_’s, ↑ chemotherapy delivery, and ↑OS in orthotopic GL261 glioma model and s.c. LLC model^j^	[+] ABTAA ↓ lung and lymph node metastases in s.c. LLC model^j^ [+] ABTAA ↓ lung metastases in spontaneous MMTV‐PyMT breast cancer model^j^
AB‐Tie1‐39	No obvious effects on immune cell infiltration^k^	[+] AB‐Tie1‐39 had modest effects on primary orthotopic mouse 4T1 breast cancer^k^	[+] Reduced lung metastasis when used in neoadjuvant treatment settings.^k^

[+], Therapeutic benefit; [○], no therapeutic benefit; [−], worsened disease; s.c., subcutaneous; OS, overall survival; LLC, Lewis lung carcinoma; LM2‐4, a highly metastatic derivative of MDA‐MB‐231 human breast cancer cell line (Munoz *et al,* 2006; Man *et al,* 2007); LNM35, a highly metastatic derivative of NCI‐H460 human lung cancer cell line; SN12, SK‐NEP‐1, human renal cancer cell lines; A321, human epidermoid cancer cell line; Colo205, HT29, human colorectal cancer cell lines; TAMs, tumor‐associated macrophages; T_reg_’s, tumor‐infiltrating regulatory T cell; ^[^ ^k^ ^]^ and ^[P]^, COMP‐Ang1 delivered by intravenously injected adenoviral vector versus protein.

^a^ Cho *et al* (2004b).

^b^ Lee *et al* (2008).

^c^ Hwang *et al* (2009).

^d^ Wu *et al* (2016b).

^e^ Holopainen *et al* (2009).

^f^ Daly *et al* (2013).

^g^ Huang *et al* (2009).

^h^ Wu *et al* (2015).

^i^ Goel *et al* (2013).

^j^ Park *et al* (2017).

^k^ Singhal *et al* (2020).

Further evidence for the potential of Ang1 supplementation to limit metastasis can be gleaned from studies using Ang1‐deficient mice. Deletion of Ang1 increased lung metastasis in both B16 melanoma and MMTV‐PyMT breast cancer models whereas primary tumor growth was unchanged (Michael *et al,* 2017). This was likely due to increases in tumor cell extravasation and adhesion to endothelium.

However, in more recent preclinical studies investigating CRC liver metastases, the opposite seems to be the case. Ang1 KO mice displayed less MC38 colon tumor experimental liver metastases generated by intra‐splenic injection compared with wild‐type controls (Ibrahim *et al,* 2019). Interestingly, the few liver metastases that did form in Ang1 KO mice were driven by angiogenesis instead of the previously observed dominant patterns of vessel co‐option‐dependent growth in wild‐type mice. Ang1 was shown to be expressed by hepatocytes and could increase the invasiveness and migratory potential of tumor cells. Therefore, caution may need to be practiced with Ang1 supplementation in cancer types that readily metastasize to the liver, as may also be the case with Ang2 blockade, discussed previously.

There are also examples where Ang1 overexpression has led to contradictory effects to those mentioned previously. In one study, intravenous delivery of adenoviral vectors was used to induce overexpression of Ang1 to supraphysiological levels and this promoted lung metastasis from subcutaneously grown or tail vein‐injected LMN35 human lung cancer cells (Holopainen *et al,* 2009). Such differences could be tumor model dependent or indeed could be due to ectopic overexpression of Ang1 to supraphysiological levels, which is likely quite different from supplementing with Ang1 mimetic drugs. Furthermore, outcomes in these studies may have been different if chemotherapy was included in the Ang1 overexpression/ supplementation studies. Ang1 and Tie2 activation both have the potential to improve intratumoral drug delivery, and this may be responsible for at least some of their beneficial anti‐tumor effects in adjuvant therapy settings (Fig. 5B).

### *Perioperative* *use of “Vascular Stabilizers”: Tie2 agonists*

Beyond engineered Ang1 variants, there are now alternative Tie2 activators in the form of AKB‐9778, ABTAA, or AB‐Tie1‐39 (Table 3). Preclinically, AKB‐9778 (a VE‐PTP inhibitor) has shown Tie2‐activating activity and therapeutic potential in the adjuvant setting—alone or in combination with chemotherapy—in a syngeneic model of spontaneous lung metastases occurring after resection of orthotopic 4T1 murine breast tumors (Goel *et al,* 2013). The anti‐tumor effects of ABTAA (an Ang2 oligomerizing antibody that activates Tie2) were recently evaluated in several mouse tumor models (Park *et al,* 2017); its anti‐metastatic potential to lymph nodes and lung were also shown but only in the presence of unresected primary tumors (Table 4). It remains to be determined whether ABTAA would also be effective if examined explicitly as a perioperative or neo/adjuvant treatment in preclinical models. Indeed, the Tie1‐specific antibody AB‐Tie1‐39 (which activates Tie2 *in vivo*) could protect mice from lung metastases when used in a neoadjuvant treatment setting before primary tumor resection (Singhal *et al,* 2020). These findings somewhat recapitulate previous studies involving mice with inducible endothelial Tie1 deficiency, which displayed decreased metastasis due to prevention of tumor cell extravasation (La Porta *et al,* 2018). These studies reinforce that activating Tie2 may act to suppress or prevent early metastatic cell dissemination events (Fig. 5C).

There are still many questions when it comes to the use of Ang1 supplementation and Tie2 activation. For example, it is still unclear what effect this therapeutic strategy will have on pro‐tumor and pro‐metastatic Tie2‐expressing monocytes and macrophages. Additionally, and as mentioned previously, such strategies may in fact have pro‐metastasis effects in some tumor types, such as those that metastasize to the liver. Additional studies are needed to help clarify these issues.

## Current and future therapeutic investigations

### *Clinical* *developments targeting the Ang/Tie axis*

The McCAVE phase II clinical study investigated the use of vanucizumab (a bispecific VEGF and Ang2 blocking antibody) in patients with metastatic CRC. This trial failed to show an advantage for vanucizumab over bevacizumab when administered with chemotherapy using a modified FOLFOX6 protocol; this led to the discontinuation of trials investigating vanucizumab monotherapy (Bendell *et al,* 2020). Nonetheless, there are ongoing clinical trials investigating the use of vanucizumab in combination with atezolizumab (anti‐PD‐L1) or CD40 agonists (Table 2), although atezolizumab plus vanucizumab has thus far not shown significant benefit over the monotherapies in platinum‐resistant recurrent ovarian cancer (Oaknin *et al,* 2017) (Table 2).

### *Combining* *Ang2/VEGF co‐targeting strategies with anti‐PD‐1/PD‐L1 immune checkpoint antibodies in the adjuvant setting: a potentially promising therapy?*

With the goal of evaluating the adjuvant use of immune checkpoint inhibitors that target the PD‐1/PD‐L1 pathway, multiple phase III clinical trials have been initiated or are currently recruiting patients (www.clinicaltrials.gov) — including several designed to test nivolumab (anti‐PD‐1 antibody) in the adjuvant setting for resected gastroesophageal cancer (NCT02743494), resected non‐small cell lung cancer (NSCLC) (NCT02595944), resected urothelial carcinoma (NCT02632409) which recently reported positive results (Bajorin *et al,* 2017), resected melanoma (NCT03068455), resectable RCC (NCT03055013; NCT03138512); as well as several trials designed to test atezolizumab (anti‐PD‐L1 antibody) in resected urothelial carcinoma (NCT02450331), resected RCC (NCT03024996), and resected NSCLC (NCT02486718).

An important question is whether the addition of Ang2/VEGF‐, Tie1‐, or Tie2‐targeted agents might improve the efficacy of adjuvant anti‐PD‐1/PD‐L1‐based therapies in the perioperative setting to treat micrometastatic disease. Some evidence does suggest that therapies targeting the Ang/Tie pathway may play a relevant role in improving immunotherapy outcomes. For instance, treatment‐associated increases in serum Ang2 appeared to predict reduced OS and reduced responses to CTLA‐4/PD‐1 blockade in patients with advanced melanoma (Wu *et al,* 2017). There is also accumulating preclinical evidence showing improved therapeutic benefits when Ang2/VEGF pathway inhibitors are combined with PD‐1/PD‐L1 pathway inhibitors in mice to treat various primary tumors (Schmittnaegel & De Palma, 2017; Schmittnaegel *et al,* 2017; Tacchio *et al,* 2019). Interestingly, treatment with VEGFR2‐specific, VEGF‐specific, or VEGF/Ang2‐bispecific antibodies led to increased proportions of PD‐L1‐expressing intratumoral endothelial cells in several of the aforementioned preclinical models (Allen *et al,* 2017; Schmittnaegel *et al,* 2017). Additionally, Ang2 has been shown to upregulate PD‐L1 surface expression on Tie2^+^ M2‐polarized macrophages (Wu *et al,* 2017). It remains to be determined whether these treatments—when applied to the perioperative setting—will have similar effects of modulating PD‐L1 expression on endothelial cells or macrophages and what implications there may be for therapeutic control of postsurgical early‐stage disease. The use of VEGF/Ang2 inhibition has also been demonstrated to be a potential combination partner with agonist CD40 antibody immunotherapy (Kashyap *et al,* 2020).

Given the aforementioned information, and some additional considerations, it is reasonable to make a strong case or rationale for using drugs that target Ang2 or modulate (activate) Tie2 in combination with immune checkpoint antibodies in the postsurgical adjuvant setting. Overall, the rationale can be summarized as follows. First, there have been a number of positive randomized phase III clinical trials involving either a PD‐1 or PD‐L1 antibody in combination with a VEGF pathway targeting anti‐angiogenic agent. These successes include the anti‐angiogenic TKI, axitinib, in combination with either pembrolizumab or the PD‐L1 antibody known as avelumab, in advanced renal cell carcinoma (RCC) (Motzer *et al,* 2019; Rini *et al,* 2019), the TKI cabozantinib in combination with nivolumab in RCC (Choueiri *et al,* 2020), and the TKI lenvatinib in combination with pembrolizumab in RCC (Motzer *et al,* 2021), as well as the PD‐L1 antibody, atezolizumab, in combination with bevacizumab for the treatment of advanced hepatocellular carcinoma (Finn *et al,* 2020), and the combination of atezolizumab, bevacizumab, and carboplatin in metastatic non‐squamous NSCLC (Socinski *et al,* 2018). Second, in contrast to a variety of anti‐angiogenic drugs, which as summarized earlier, have uniformly failed in numerous phase III clinical trials in the adjuvant setting, the situation appears more promising regarding the use of immune checkpoint antibodies in the same adjuvant treatment setting. Already there is one such phase III clinical trial success that has led to a marketed approval, i.e., the use of ipilimumab, the CTLA‐4 targeting antibody, for the treatment of postsurgical adjuvant high‐risk melanoma patients (Barker, 2018; Eggermont *et al,* 2018a, 2018b; Helmink *et al,* 2018). This initial success has generated enthusiasm for undertaking many similar adjuvant trials in other indications which are currently underway, for example, in non‐small‐cell lung cancer patients (Vansteenkiste *et al,* 2019). Given these various clinical trial results, there would normally be a rationale for testing a VEGF pathway inhibiting drug with an immune checkpoint antibody in the adjuvant setting, but this is clearly problematic given the many phase III trial failures of various anti‐angiogenic drugs in the adjuvant treatment setting, as summarized earlier. However, this may not be the case for the rationale of combining a drug which modulates Ang2, Tie2, or Tie1 for all the reasons discussed here.

### *Ang‐Tie* *modulation and immunotherapy in the neoadjuvant treatment setting?*

The use of Ang‐Tie pathway modulation strategies to stabilize the vasculature to inhibit metastasis would of course only be viable in early‐stage cancers where the tumor cells have not already disseminated at the time of such treatments. In this context, the use of neoadjuvant therapies may be the most important (Gengenbacher *et al,* 2020; Singhal *et al,* 2020), if indeed patients can be treated at a time point before the metastatic cascade has taken place. There is evidence to suggest that early on in tumor development some cancer types may have already disseminated (Eyles *et al,* 2010; Phan & Croucher, 2020). However, the benefit of neoadjuvant regimens using targeted therapies or immunotherapies has recently been highlighted in melanoma patients with resectable stage III disease, where it was shown that if patients displayed a pathological complete response to therapy in the neoadjuvant setting this correlated well with improved recurrence‐free survival and OS 2 years post‐surgery (Menzies *et al,* 2021). There are also positive results reported from clinical trials in which an immune checkpoint inhibitor is used in the neoadjuvant setting in TNBC patients (Mittendorf *et al*, 2020). One potential caveat to neoadjuvant immunotherapy is the progression of disease and metastatic dissemination during the pre‐surgical treatment window. The use of immunotherapy along with agents that can block the metastatic process such as those discussed in this review may have substantial benefits in increasing the proportion of patients who initially respond and then have an improved chance of displaying long‐term survival benefits. This two‐pronged approach in expanding the pool of tumor‐reactive T cells and subsequent enhancement of immune memory formation by use of immunotherapy (Topalian *et al,* 2020), coupled with limiting the metastatic process, has potential clinical value, if indeed it does not increase the rate of severe toxicity in patients.

## Conclusion

The clinical testing of anti‐Ang2 therapeutics has thus far been limited to evaluation in late‐stage disease settings, and these studies have shown disappointing results. There is, however, preclinical evidence to suggest that agents which modulate Ang2 function or Tie1/Tie2 receptors may have more promise in the perioperative neoadjuvant or adjuvant therapy setting to treat or inhibit early‐stage micrometastatic disease. Potential caveats to this approach include the possibility of Ang2 blockade or Tie2 activation enhancing liver metastases, the opposite of what is observed in lung metastases (Im *et al,* 2013; Ibrahim *et al,* 2019). More experimental validation using preclinical models that display metastasis to different organ sites other than the lung will need to be performed with these Ang/Tie modulating agents. In conclusion, future investigations combining Ang/Tie modulatory agents with other therapies such as VEGF/VEGFR2 pathway inhibitors, conventional (or metronomic) chemotherapy, or indeed immunotherapy may be worthy of further preclinical validation and future clinical trial evaluation for the treatment of early‐stage disease.

## Conflict of interest

RSK is a member of the Scientific Advisory Board of Angiocrine Bioscience Inc., CSTS Healthcare, Novelty Nobility, Nonagen Therapeutics and OncoHost and a consultant to Novelty Nobility, Pharmabcine, and CSTS Healthcare. The other authors declare no conflicts of interest.

**Pending issues**

1. Can Ang2 inhibitors and/or Tie2 activators be used successfully in the clinical prevention and/or treatment of early‐stage cancer, i.e., in the neoadjuvant or adjuvant therapy settings?
2. Does Ang2 inhibition or Tie2 activation have differential effects on metastasis depending on the organ in question (i.e., lung vs. liver)?
3. Can Ang2 inhibitors or Tie2 activators be successfully combined with immunotherapy in the clinical treatment of early‐stage cancer?
4. Will pharmaceutical companies consider evaluating therapeutic agents in phase III neoadjuvant (or even adjuvant trials) in the absence of positive phase III clinical trial data in the advanced metastasis therapy settings—when there is strong evidence to explain why such therapies would not or did not work in late‐stage disease settings?
